# A review of neovascular glaucoma. Etiopathogenesis and treatment

**DOI:** 10.22336/rjo.2021.66

**Published:** 2021

**Authors:** Lilia Dumbrăveanu, Valeriu Cușnir, Doina Bobescu

**Affiliations:** * Department of Ophthalmology and Optometry, “Nicolae Testemițanu” State University of Medicine and Pharmacy, Chișinău, Republic of Moldova

**Keywords:** neovascular glaucoma, ocular ischemia, angiogenesis, anti-VEGF, glaucoma surgery

## Abstract

Neovascular glaucoma (NVG) is a type of secondary glaucoma, refractory to treatment, often incurable, with very poor visual prognosis. It is characterized by the appearance of new vessels over the iris and iridocorneal angle and frequently associates the presence of a fibrovascular membrane which limits the aqueous humor outflow from the anterior chamber. The most common causes of NVG are: central retinal vein occlusion, proliferative diabetic retinopathy, and ocular ischemic syndrome. Once the gonioscopy developed as a part of clinical examination, it became possible to visualize the new vessels of the anterior segment of the eye in early stages and to understand the mechanisms of increased intraocular pressure (IOP), including narrowing and closing of the iridocorneal angle. Also, the modern imaging techniques, such as optical coherence tomography angiography and fluorescein angiography became indispensable for the clinician. Thus, an early diagnosis, followed by starting an appropriate therapy: panretinal photocoagulation or administration of anti-VEGF drugs, with or without hypotensive ocular therapy, allows the preservation of visual functions for patient’s better quality of life. However, one or more surgeries will often be required, especially in the advanced stages of the disease, which do not respond to drug therapy. Managing the NVG we should aim to: 1) reduce ocular ischemia and treat its underlying cause, 2) reduce elevated IOP, once installed and 3) control the inflammatory process. Anyway, the best treatment is prevention, so we must be very attentive at patients with risk factors for developing the NVG.

**Abbreviations:** NVG = neovascular glaucoma, ICA = iridocorneal angle, IOP = intraocular pressure, TM = trabecular meshwork, AH = aqueous humor, AC = anterior chamber, PRP = panretinal photocoagulation, VEGF = vascular endothelial growing factor, Anti-VEGF = anti- vascular endothelial growing factor, PAS = peripheral anterior synechiae, CRVO = central retinal vein occlusion, PDR = proliferative diabetic retinopathy, DR = diabetic retinopathy, OIS = ocular ischemic syndrome, CRAO = central retinal artery occlusion, ROP = retinopathy of prematurity, FEVR = familial exudative vitreoretinopathy, PVR = proliferative vitreoretinopathy, MMPs = matrix metalloproteinases, VEGFR = vascular endothelial growing factor receptor, PDGF = platelet-derived growth factor, PIGF = placental growth factor, NRP = neuropilins, HIF = hypoxia-inducible factor, SDF1 = stromal cell-derived factor 1, DDL4 = delta like ligand 4, NICD = Notch intracellular domain, TIMMPs = tissue inhibitors of matrix metalloproteinases, ANGPT = angiopoietin, Tie 2 = tyrosine-protein kinase receptor for angiopoietins, IGF-1 = insulin-like growth factor 1, RPE = retinal pigment epithelium, IL = interleukin, TNF = tumor necrosis factor, bFGF = basic fibroblast growth factor, TGF = transforming growth factor, HGF = hepatocyte growth factor, TNFR 2 = tumor necrosis factor receptor 2, OIR = oxygen induced retinopathy, NVI = neovascularization of the iris, NVA = neovascularization of the iridocorneal angle, FA = fluorescein angiography, RAPD = relative afferent pupillary defect, CNP = capillary non-perfusion, NVE = neovascularization elsewhere in the retina, NVD = neovascularization of the optic disc, FFA = fundus fluorescein angiography, OCTA = optical coherence tomography angiography, B-scan US = B-scan ocular ultrasound, AS-OCT = anterior segment optical coherence tomography, ARC = anterior retinal cryotherapy, FDA = food and drug administration, United States of America, BVZ = bevacizumab, RBZ = ranibizumab, AFB = aflibercept, AMD/ ARMD = age related macular degeneration, DME = diabetic macular edema, GDDs = glaucoma drainage devices, MMC = mitomycin C, 5-FU = 5-fluorouracil, AGV = Ahmed glaucoma valve, AADI = Aurolab aqueous drainage implant, MIGS = minimally invasive glaucoma surgery, BCVA = best corrected visual acuity, TVT = Tube versus Trabeculectomy study, MPC = micro-pulse cyclophotocoagulation.

## Introduction

Neovascular glaucoma (NVG) represents a type of secondary glaucoma, which in 95% of cases appears as a result of retinal ischemia. NVG associates the iris and iridocorneal angle (ICA) rubeosis, connective tissue growth, elevated intraocular pressure (IOP) and is often refractory to drug and surgical therapies. As a result, NVG became a sight-threatening condition, with high rates of severe visual acuity impairment, being potentially blinding and very often incurable.

Any cause of retinal ischemia triggers the mechanism and starts to produce local angiogenic stimuli. These allow the development of a fibrovascular membrane, which blocks the trabecular meshwork (TM) and obstructs the aqueous humor (AH) outflow from the anterior chamber (AC). Initially, NVG manifests as a secondary open-angle glaucoma, which partially responds to topical ocular hypotensive drugs, panretinal photocoagulation (PRP) and anti-VEGF remedies. Later, the myofibroblasts from the new formed connective tissue and the new vessels start to contract, in this way allowing the adhesion between iris and cornea, creating peripheral anterior synechiae (PAS). The number of PAS increases gradually and tend to close the ICA. That is why the IOP rises markedly and no medication could lower it. In this situation, an antiglaucomatous surgery may be useful. Because of the complicated evolution, it is imperative to diagnose the neovascular glaucoma as early as possible and to initiate all necessary therapeutical measures, thus, just in this way, visual functions could be preserved in these patients.

The histopathological aspects of new vessels were described for the first time by Coats in 1906, in a patient with central retinal vein occlusion (CRVO). In 1928, Salus reported the existence of similar blood vessels in the iris in several patients with diabetes mellitus. In 1963, Weiss et al. proposed the term “neovascular glaucoma” and described a severe clinical form of glaucoma associated with new vessels of the iris and iridocorneal angle, connective tissue in these structures, and increased intraocular pressure.

Unfortunately, even nowadays, in the technology and scientific era, we do not have sufficient information to fully understand the pathophysiology and molecular mechanisms of action in NVG. For this reason, we are limited in having an efficient therapeutic approach in these patients, who are doomed to live in a complicated way, with low vision functions and low quality of life. Therefore, neovascular glaucoma remains a disastrous ocular pathology that does not respond to standard drug or surgical therapies and tends to end with limited visual functions and unbearable pain. The rate of severe visual impairment is very high in these patients, whose vision in the late stages of disease is just hand motion or perception of light [**1**-**8**]. 

This review article was designed to carefully study the neovascular glaucoma and to find its causes, trying to understand its pathophysiology as much as possible. Also, we had to present all known methods of treatment and generate interest for future development of new therapies, in hope of giving a better outcome to patients with NVG.

*Epidemiology*

Neovascular glaucoma represents about 3,9% in the structure of glaucomatous pathology. Even if its prevalence in the general population is low, NVG leads to a drastic decrease in visual acuity and ends up with disability. An increased prevalence was found among the elderly population. The European Union has estimated that about 75,000 to 113,000 people suffer from neovascular glaucoma, and the annual incidence is about 3,800 new cases.

It is important to note that about 60% of patients with ischemic central retinal vein occlusion present anterior segment new vessels in terms of some weeks to 1-2 years from the moment of vascular accident. 40-45% of those with CRVO will develop NVG and in 80% of them, NVG will develop in just 6 to 8 months.

65% of patients with proliferative diabetic retinopathy (PDR) will develop iris neovascularization and neovascular glaucoma will appear in 20% of them. If a diabetic patient has NVG in one eye, then his risk of developing NVG in the fellow eye is about 33%. Some data suggest any intraocular surgery, cataract extraction or vitrectomy, or others, could accelerate the progression of neovascular glaucoma in patients with PDR [**4**,**9**-**11**].

*Etiology*

Neovascular glaucoma is a tremendous ocular condition that can develop in different ocular and systemic diseases. 

Among **ocular ischemic conditions**, leading to NVG, are: central retinal vein occlusion, diabetic retinopathy (DR), carotid insufficiency or ocular ischemic syndrome (OIS), sickle cell retinopathy, radiation retinopathy, central retinal artery occlusion (CRAO), retinopathy of prematurity (ROP), familial exudative vitreoretinopathy (FEVR), persistent hyperplastic primary vitreous.

**Inflammatory** predisposing causes of NVG are: uveitis, trauma, Eales disease, retinal vasculitis, anterior segment ischemia (may be post-surgical), endophthalmitis and extraocular inflammatory vascular causes (like Giant Cell Arteritis, temporal arteritis).

**Retinal conditions** are the following: long standing retinal detachment, proliferative vitreoretinopathy (PVR), Coats disease, retinoschisis, detachment associated with intraocular tumors.

Among **tumors**, the following can be mentioned: choroidal melanoma, iris melanoma, retinoblastoma, intraocular metastasis, ciliary body medulloepithelioma, vasoproliferative tumors of the retina, hyperviscosity syndromes and myeloproliferative disorders.

**Systemic conditions** most commonly met are: juvenile myelomonocytic leukemia, systemic lupus erythematosus, juvenile xanthogranuloma, cryoglobulinemia type 1, neurofibromatosis type 1, extraocular vascular disorders like internal carotid artery obstruction and carotid cavernous fistula.

As we have listed, there are a lot of diseases that predispose and promote the appearance and development of neovascular glaucoma, but just three of them have a major role and a high frequency, namely: diabetic retinopathy (33%), ischemic central retinal vein occlusion (33%) and ocular ischemic syndrome (13%) [**2**,**5**,**12**,**13**].

## Pathogenesis

*Angiogenesis and vasculogenesis*

Despite the progress of the medical world, we are still in a state of confusion when confronting the neovascular glaucoma. That is because we have insufficient information about molecular interaction and its effects, as well as the NVG mechanism of action. Even if there are about forty different causes predisposing to NVG, all of them have a common pathway of generating endogenous factors, which are transported into the anterior segment of the eye and there they disturb the ocular homeostasis and induce specific changes. In this context, it is imperative to discuss about the vascular endothelial growing factor, as it was recognized as the most important in the genesis of the new vessels.

**Fig. 1:**
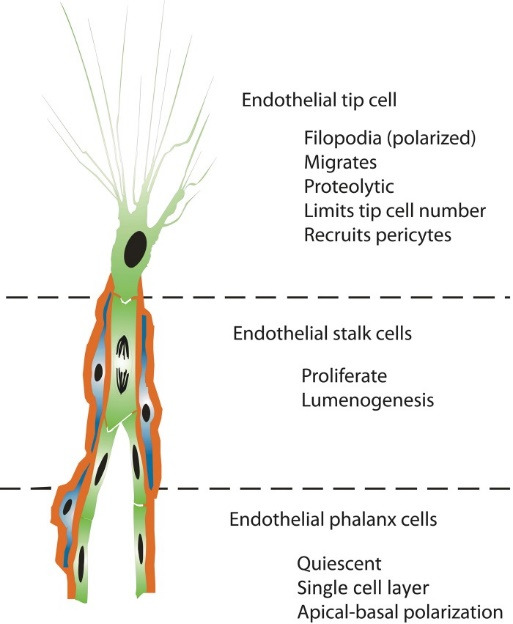
Concept of endothelial cell differentiation during angiogenesis. Angiogenic sprouts are formed by a subset of specialized endothelial cell phenotypes (green), each with a distinct cellular fate. Pericytes (blue) are instantly recruited to unsheathe the sprouting vessel and to produce a basal lamina (red) [**14**]

Angiogenesis represents the growth of new blood vessels from the existing vasculature. As a result, a new functional vascular bed is formed. Blood vessel proliferation is a physiological process for tissue growth and development. It begins in utero and continues all the lifetime, both in healthy and sick tissues. For example, in an adult, angiogenesis occurs in different stages of development of ovarian follicles, in corpus luteum during ovulation, in various reparative body processes like wound and fracture healing. At the same time, angiogenesis plays a very important role in tumors’ vascularization, in proliferative diabetic retinopathy, central retinal vein occlusion and others. Like most of the processes, angiogenesis depends on the balance between the stimulatory and inhibitory factors. It has been demonstrated that VEGF and its receptors have a decisive role for endothelial cells proliferation and migration, thus creating a base for the new vessels. The VEGF-depending signaling pathway is also important for the development of the embryonic vascular system. Of course, neovascularization is a very complex mechanism, with lots of successive stages. Some of them are: the synthesis of pro-angiogenic factors in the tissues affected by hypoxia, these factors being capable of stimulating the existing blood vessels and activating the endothelial cells; then, the endothelium releases proteolytic enzymes like matrix metalloproteinases (MMPs - we know about 26 members of this family of proteases and it has been proven that MMP 2, 9, and 14 play an important role in angiogenesis). According to their name, MMPs are responsible for adjacent blood vessels basal membrane degradation and then is the moment for the tip cells to appear from the endothelial cells of existing vessels. Afterwards, the tip cells will pave the way for the new blood vessels. It is still unclear how these tip cells are selected and what force guides them to sprout, but it has been shown that VEGF gradient influences this process. This way, endothelial tip cells become the leading cells at the tips of vascular sprouts and act for angiogenesis progression. If there is an excessive number of tip cells, then the new blood vessels will have a chaotic arrangement. That is why, vessels have a characteristic aspect in different proliferative retinopathies. At present, tip cells have a main, hierarchic role when conducting the formation of the stalk of the sprouting vessel. Tip cells have long, polarized filopodia, migrate into the extracellular matrix and receive attractive and repulsive signals for their guidance. There is no evidence that tip cells can proliferate, they do not form the vessel lumen, but are responsible for non-vascular cells recruitment, including pericytes. The very next endothelial cells, which follow the migrating tip cell, differentiate into stalk cells. Stalk cells can proliferate and will promote the lumenogenesis (creating the blood vessel lumen). Endothelial cells following the stalk cells differentiate into phalanx cells (**Fig. 1**). 

So, these three types of endothelial cells will move in a specific direction, being guided by angiogenic stimuli. When two sprouts come together, they create an anastomosis and a new blood vessel in born. The anastomosis is mediated by tissue-resident macrophages. It is very important that the new vessel is covered by functional pericytes, because a defective recruitment of these cells is bad for tissues, especially for the eye, recruitment of pericytes being a key step in angiogenesis. Once the neovessels work and supply the tissues affected by hypoxia and lack of nutrients, they can go into a rest state. Moreover, angiogenesis also depends on the VEGF-DLL4-Notch signaling pathway [**14**-**17**]. 

*VEGF and VEGFRs*

The vascular endothelial growth factor family represents key molecules for initiation and direction of blood vessel sprouting.

VEGF derives from a supergene family of platelet derived growth factor (PDGF). All VEGF are cytokines (signaling proteins) and can stimulate both angiogenesis and vasculogenesis (de novo development of the embryonic vascular system). In physiological conditions, VEGF is responsible for embryonic vascular development, creating collateral vessels after trauma or in muscular tissue when it is highly used during physical effort, or if there are some blocked blood vessels. In pathological conditions, VEGF ensures the growing tumors with oxygen and nutrients by increasing their vascularization, and allows them to metastasize. Until present, there are six known members of the VEGF family: VEGF-A, VEGF-B, VEGF-C, VEGF-D, VEGF-E and placental growth factor (PIGF).

VEGF-A is responsible for angiogenesis: stimulates endothelial cells migration, increases their mitosis, stimulates matrix metalloproteinases and integrin avb3 activity, stimulates migration and proliferation of astrocytes, helps to form vascular lumen, and creates fenestrations. Also, VEGF-A has a chemotactic role for macrophages and granulocytes, indirectly contributing to vasodilatation and increased blood flow, which precedes angiogenesis (by the release of nitric oxide) and participates in lymphangiogenesis. VEGF-B is responsible for embryonic angiogenesis. VEGF-C participates in lymphangiogenesis.

VEGF-D is necessary for the development of lymphatic vessels of adjacent pulmonary bronchioles.

VEGF-E was discovered in the Orf virus genome.

PIGF has an important role in vasculogenesis and angiogenesis (if there is ischemia), in inflammatory processes, wound healing and neoformations.

Each type of VEGF could bind to one or two VEGF receptors. There are three VEGFR: VEGFR1, VEGFR2, VEGFR3. All of them are trans-membrane tyrosine-kinase type receptors. VEGF-A binds to VEGFR1 (encoded by FLT1 gene) and VEGFR2 (encoded by KDR/ FLK1 gene). VEGF-B binds just to VEGFR1. VEGF-C and VEGF-D are specific ligands for VEGFR3 (encoded by FLT4 gene) and this way are responsible for embryonic angiogenesis and lymphangiogenesis. Also, VEGF-C and VEGF-D bind to VEGFR2 and can stimulate angiogenesis. VEGF-E binds just to VEGFR2 and PIGF binds just to VEGFR1, which acts for embryonic vasculogenesis. Anyway, the activation of VEGFR2 starts most cellular responses to VEGF. Beside its main role, VEGFR1 can modulate VEGFR2 signaling by sequestering VEGF from VEGFR2. In addition to binding VEGF to VEGFRs, vascular endothelial growth factors may also bind to receptor complexes, which are composed of VEGFRs and neuropilins. This receptor complex emits stronger signaling for VEGF in endothelial cells. Neuropilins (NRP-1 and NRP-2) are active protein receptors in neurons, capable of regulating neurogenesis and angiogenesis. They act mainly as co-receptors, due to their pleiotropic nature.

Retinal hypoxia is a crucial moment for the synthesis and elimination of hypoxia-inducible factor (HIF-1 and HIF-2), which stimulates the expression of VEGF, platelet-derived growth factor B (PDGF-B), placental growth factor, stromal cell-derived factor 1 (SDF1) with its receptors and angiopoietin 2. That is why a new therapeutic trend has appeared, aiming at the inhibition of HIF-1 by gene engineering techniques and inhibition of PDGF-B, respectively, an additional therapeutic target. Theoretically, each pathogenetic level is a potential target for treatment, but it is very difficult to achieve in practice [**14**-**21**].

*VEGF-DLL4-Notch signaling pathway*

VEGF acts like a chemotactic agent for endothelial tip cells. VEGFR2, VEGFR3 and NRP-1 co-receptors are expressed on their surface. Once they meet, the ligand (VEGF) binds to its receptors and activates tip cells. Then, they will express the delta like ligand 4 (DLL4, encoded by DLL4 gene). Later, DDL4 binds as a ligand to the Notch receptors (Notch 1) from the surface of endothelial stalk cells that follow their tip cells. DLL4-Notch interaction results in the clivage of Notch protein by a stalk cell gamma secretase, this way releasing an intracellular domain of Notch protein (NICD). NICD reduces VEGFR2, VEGFR3 and NRP1/ 2 expression. Also, it can stimulate VEGFR1 expression in stalk cells. When VEGF binds to VEGFR1, the whole VEGF signaling pathway is blocked.

Another critical component is Jagged 1 (a protein encoded by JAG1 gene) with a major role in intercellular signaling. It is highly expressed in endothelial stalk cells and acts as an antagonist for DLL4, blocking the Notch pathway. Because the Notch protein is present both in the stalk and tip endothelial cells, JAG1 can also bind to the Notch from the tip cell, this way inactivating the signaling [**14**,**16**,**17**,**21**,**22**].

*Pericyte recruitment*

Pericytes are indispensable cells for new vessels survival. They can stimulate the migration of endothelial cells by expressing proteases and proteoglycans at the top of the vascular sprout and facilitate the endothelial tip cells motility. The interaction between endothelial cells and pericytes lead to synthesis of MMPs, especially MMP 14. Once the neovessels are formed, they need to be stabilized and pericytes are responsible for this function. When pericytes interact with endothelial cells, MMPs tissue inhibitors (TIMMPs) are secreted to reduce the proteolysis and stabilize the vessels.

The pericyte recruitment takes place under PDGF-B, angiopoietins 1 and 2 (ANGPT1 and ANGPT2) and their receptor (Tie2) control. PDGF-B is produced by the buds of vascular sprout. Its gradient promotes the pericyte recruitment. Tie 2 is a tyrosine-protein kinase receptor for angiopoietins, which regulates angiogenesis, endothelial cell survival, proliferation, migration, adhesion, and cell spreading, reorganization of the actin cytoskeleton, but also maintenance of vascular quiescence. Tie 2 can activate or inhibit angiogenesis, depending on the context. For example, ANGPT1 derived from pericytes can activate Tie 2, this way stimulating the adhesion of pericytes to the capillary wall. ANGPT2 has an antagonist effect on Tie 2 and represents an important marker for endothelial tip cells. High VEGF and ANGPT2 levels are associated with a high rate of blood vessel destabilization, endothelial proliferation and pericyte activation [**17**, **21**-**24**].

*Other factors*

There are some types of cells in the eye that can secrete vascular endothelial growth factor and insulin-like growth factor 1 (IGF-1). These are: Muller cells, retinal pigment epithelium (RPE), pericytes of the retinal capillaries, endothelial and ganglion cells, and ciliary non-pigmented epithelium. It has been proven that VEGF is not enough for angiogenesis and IGF-1 should also be present in the aqueous humor. Then, the rubeosis of the iris and peripheral anterior synechia will appear and will block the iridocorneal angle and AH outflow.

In patients with neovascular glaucoma, the ciliary non-pigmented epithelium is the main place of VEGF synthesis. That is why it has become a new therapeutical target for PRP non-responders.

Another pro-angiogenic factor is interleukin-6 (IL-6). In NVG secondary to CRVO, the level of IL-6 in AH correlates with the degree of iris neovascularization. In patients with proliferative diabetic retinopathy, there is a high concentration of interleukin-1 (IL-1), which activates the lymphocytes, IL-8 (with chemotactic role on neutrophils), IL-18 (activates macrophages) and tumor necrosis factor alpha (TNF alpha). Cases of tumor necrosis factor receptor 2 (TNFR2) activation, whose signaling may be an ideal strategy for the treatment of oxygen-induced retinopathy (OIR) model, are described in literature. There is some evidence of basic fibroblast growth factor (bFGF) implication in the pathogenesis of NVG. In addition, high concentrations of transforming growth factor beta 1 (TGF-beta 1), hepatocyte growth factor (HGF), nitric oxide, endothelin 1 and superoxide free radicals were found in the AH of NVG patients. Each of these factors serves as a potential target for NVG therapy and further studies are needed to evolve new strategies [**14**,**23**-**33**].

*Classification of iris and iridocorneal angle neovascularization*

Wand described the following 4 stages:

1. Incipient rubeosis iridis that is characterized by the appearance of dilated capillaries at the pupillary margin. The iridocorneal angle is not affected.

2. Moderate rubeosis iridis - the neovessels are arranged radially, towards the periphery of the iris. At the iris collarette level is present a dilated circumferential vessel. ICA is not affected. 

3. Advanced rubeosis iridis – the new vessels appear in the ICA. They come from the great arterial circle of the iris, cross the scleral spur and branch at the level of the trabecular meshwork. PAS appear in ICA.

4. Neovascular glaucoma – ICA is closed at 360 degrees by the PAS, the pupil is deformed and an ectropion uveae may appear [**30**].

Weiss and Gold proposed a four-grade classification for iris and iridocorneal angle rubeosis (**Table 1**):

**Tabel 1:** Weiss and Gold classification of iris and iridocorneal angle neovascularization [**29**].

	Grade 1	Grade 2	Grade 3	Grade 4
Neovascularization of the iris (NVI)	Fine surface neovascularization of the pupillary zone of the iris involving <2 quadrants	Surface neovascularization of the pupillary zone of the iris involving more than 2 quadrants	In addition to neovascularization of the pupillary zone, neovascularization of the ciliary zone of the iris and/ or ectropion uveae involving 1-3 quadrants	Neovascularization of the ciliary zone of the iris and/ or ectropion uveae involving more than 3 quadrants
Neovascularization of the iridocorneal angle (NVA)	Fine neovascular twigs cross scleral spur and ramify on trabecular meshwork involving <2 quadrants	Neovascular twigs cross scleral spur and ramify on trabecular meshwork involving more than 2 quadrants	In addition to neovascularization of trabecular meshwork, peripheral anterior synechiae (PAS) involving 1-3 quadrants	PAS involving more than 3 quadrants

## Clinical presentation

The clinical presentation of neovascular glaucoma is quite similar even if there are a lot of causes that can lead to its development. There is a difference in disease intensity and timing of its apparition after the vascular accident.

The first visible clinical sign of NVG are iris new vessels. Initially, they appear at the pupillary zone, but may also be present in ICA. Very often, it is better to perform a fluorescein angiography (FA) at the very initial stage in all suspects, because FA will detect all leakage zones and the clinician will be able to diagnose the prerubeotic stage before the neovessels become visible at biomicroscopy.

Unlike normal blood vessels, which are radial and derive from the ciliary trunk and circular ciliary band, iridis neovessels are thin, tortuous, and chaotically oriented on the iris surface.

Another critical finding is the fibrovascular membrane. It appears as a result of connective tissue proliferation and tends to close the iridocorneal angle. Usually, it is transparent and creates diagnostic difficulties, because it is hard to be detected during gonioscopy and the ICA appears as opened even if it is totally closed.

The intraocular pressure rises gradually and correlates with the ICA closing. At this moment, IOP tend to become refractory to any medication because of the closed anterior chamber angle. Later, when the fibrovascular membrane contracts, zones where the iris and cornea contact and adhere to each other appear, creating peripheral anterior synechiae. PAS tend to multiply step by step, causing the ICA to close in a zipper pattern. The terms of PAS appearance vary and can be from 3 months to some years. Usually, the PIO rises acutely, reaching levels of 40 to 60 mmHg or even more, and associates violent pain. Moreover, there can be an anterior chamber hemorrhage and/ or inflammation. The anterior chamber inflammation and flare could be delusive and it can be confused with uveitis. The slit lamp examination will detect diffuse conjunctival vessels injection, diffuse corneal edema, iris rubeosis and ectropion uveae. Also, there may be a relative afferent pupillary defect (RAPD or APD), depending on the optic nerve and retinal impairment.

The visual acuity in patients with neovascular glaucoma is frequently limited at counting fingers and light perception.

During gonioscopy, the clinician can visualize neovessels in the iridocorneal angle and peripheral anterior synechiae that close the anterior chamber angle partially or totally. Very often, it is challenging to be performed because of the corneal edema and there is little chance to detect something in acute phases with elevated IOP. 

Fundus examination can reveal diabetic retinopathy, retinal vein occlusion or ocular ischemic syndrome. In 10% of CRVO, it is important to know that the new vessels will appear firstly in the iridocorneal angle and then in the iris, that being the reason why gonioscopy should always be performed [**1**-**4**,**9**]. 

**Table 2** presents the clinical stages of neovascular glaucoma.

**Tabel 2:** Clinical stages of neovascular glaucoma [**29**]

Stage	Iris rubeosis	Secondary open-angle glaucoma	Secondary angle-closure glaucoma
Clinical features	Tiny tufts of new vessels appear first at the pupillary margin and less commonly at the angle that crosses the scleral spur to ramify over the trabecular meshwork. New vessels grow over iris surface in an irregular fashion	Development of a fibrovascular membrane on anterior surface of the iris and iridocorneal angle, which blocks the trabecular meshwork, and obstructs aqueous humor outflow in an open-angle manner	Contracture of the fibrovascular membrane pulls the iris over the trabecular meshwork, forming peripheral anterior synechiae
Neovascularization of the iris (NVI)	Present	Prominent	Prominent, with ectropion uveae
Gonioscopy	Open iridocorneal angle NVA with or without NVI may be present	Open iridocorneal angle NVA may or may not be visible	Closed iridocorneal angle NVA usually not visible
IOP	Normal	Elevated	Elevated
Prognosis	Good	Good with timely intervention	Usually guarded

*Instrumental investigations*

The iris and iridocorneal angle neovessels may not be detected during biomicroscopy and gonioscopy. In these cases, it is crucial to perform a fluorescein angiography because it is very useful for detecting early stage rubeosis and even the prerubeotic stage. Fundus fluorescein angiography (FFA) is the gold standard for the diagnosis of capillary non-perfusion zones (CNP) and retinal neovessels (NVE and NVD). Ultrawide-field FFA allows the visualization of an extended zone of the retina of about 200 degrees. FA is very good at detecting leakage from neovessels, but its use can be restricted in allergic to dye patients. That is why the optical coherence tomography angiography (OCTA) becomes more and more actual. OCTA is a non-invasive and patient-friendly method. It is an ideal instrument for patient monitoring, fixing the neovessels regression and detecting neovascularization of the iris. It has been shown that OCTA has a 79% sensitivity and 97% specificity against FA. Wide-field OCTA has 100% sensitivity and 97% specificity against Ultrawide-field FA. The results were similar for Wide-field OCTA and Ultrawide-field FA in patients who underwent panretinal photocoagulation 3 months before the investigation. In the context of neovascular glaucoma, we should not underestimate the importance of B-scan ocular ultrasound (B-scan US). It is very informative for the exclusion of the rare and unexplained causes of NVG like long-standing retinal detachment and intraocular tumors. B-scan US is preferred in young patients, to rule out ciliary body tumors.

The wide spectrum of neovascular glaucoma causes needs to be investigated as much detailed as possible. Because of the systemic associations, NVG patients should undergo a careful approach.

Blood pressure will be measured and monitored regularly to diagnose arterial hypertension. Blood glucose levels and glycated hemoglobin (HbA1c) will be measured to detect diabetes. To exclude ocular ischemic syndrome, patients need: Doppler examination of the carotid arteries (retrobulbar, intra- and extracranial vessels), magnetic resonance imaging, computed tomography, carotid intra-arterial digital subtraction angiography (selectively and with extreme caution). Computed tomography, magnetic resonance imaging or positron emission tomography (PET) scanning will be performed to exclude carotid-cavernous fistula and tumor metastases. The HLA B27 complex will be tested for uveitis, retinal vasculitis, and blood dyscrasias. In addition to all these investigations, the hemogram with erythrocyte sedimentation rate, C-reactive protein, antinuclear antibodies, lipid profile, Wasserman reaction, and serum proteins will be tested. Specific investigations will be performed to rule out tuberculosis, sarcoidosis, and blood dyscrasias [**2**,**34**-**39**].

*Differential diagnosis*

Differential diagnosis can be divided into etiologies of true neovascular glaucoma (which were fully described before in the NVG „Etiology” section) and other ocular conditions that can clinically mimic the presentation of NVG. These are summarized in the next table (**Table 3**).

**Tabel 3:** Conditions that can clinically mimic the presentation of neovascular glaucoma [**2**,**5**,**12**,**13**,**40**]

Ocular condition	Differentiating features	Ancillary investigations
Uveitis	Engorged iris blood vessels, keratic precipitates, anterior chamber cells	Slit lamp, uveitis workup, blood tests
Acute angle-closure glaucoma	Shallow anterior chamber, closed angles, convex iris	Slit lamp, gonioscopy, anterior segment-OCT (AS-OCT), fundoscopy, fellow eye examination
Chronic angle-closure glaucoma	Shallow anterior chamber, closed angles, convex iris configuration, pupillary block, NVI; no NVE/ NVD	Slit lamp, gonioscopy, AS-OCT, fundoscopy
Intraocular tumors	Neovascularization of iris and iridocorneal angle ±	Slit lamp, fundus examination, B-scan USG, ancillary imaging for metastasis
Carotid-cavernous fistula	Blood in Schlemm’s canal	Gonioscopy, imaging investigations of brain
Anterior segment dysgenesis	Corectopia, iris atrophy with prominent blood vessels	Gonioscopy, fundoscopy
Retinal detachment	If longstanding, proliferative vitreoretinopathy changes and neovascularization	Biomicroscopy, fundoscopy, B-scan USG

We should always be aware of normal light-colored eyes with prominent iris vessels, not to confound them with neovessels.

In cases of anterior uveitis, especially after surgery, the engorged iris blood vessels and/ or ocular hypotonia may be confused with NVG. For differentiation, corticosteroids are instilled topically, as they lead to constriction of the blood vessels, unlike it happens in cases of NVG, where the iris neovessels do not regress.

Acute angle-closure glaucoma mimics the neovascular glaucoma in the best way. Usually, patients come with acute ocular pain, decreased visual acuity, elevated IOP and dilation of circumciliary blood vessels. All these findings may be delusive, because the clinician may interpret them as iris neovessels. In cases of corneal edema, when the biomicroscopy and fundoscopy are irrelevant, we should carefully examine the fellow eye, its iridocorneal angle and fundus appearance, where we could find signs of retinal hypoxia that sustain the diagnosis of neovascular glaucoma. Patients with ocular ischemic syndrome due to obstruction of the carotid artery have a history of ocular pain, amaurosis fugax and metamorphopsia. Their IOP may be both normal and low, even after the NVG has established.

In anterior segment dysgenesis, especially essential iris atrophy, in Fuchs’ heterochromic iridocyclitis and pseudoexfoliation syndrome, it is common to find the anterior segment neovascularization and elevated intraocular pressure.

Every time during clinical examination, it is very important to differentiate prominent iris vessels from iris rubeosis. In the case of prominent iris vessels, they are always radial, lie within the iris stroma and never traverse the scleral spur. In contrast, the neovessels are chaotically arranged, lie superficial and often cross the scleral spur. As a result, the iridocorneal angle closes in a zipper pattern [**2**,**5**,**13**,**40**-**43**].

*Treatment*

The treatment of neovascular glaucoma is a real challenge both for the clinician and the patient. Unfortunately, very often, even the necessary therapy is applied in a correct way and in good terms, the patient’s outcome and visual prognosis are poor and his life quality is affected severely. The clinical practice shows the importance of some treatment principles. First, we should treat the retinal ischemia, thus reducing the release of pro-angiogenic stimuli. This step can be done by injecting anti-VEGF agents into the vitreous body or by performing the panretinal photocoagulation. Next principle considers to treat the underlying systemic disease, this way balancing the blood flow to the eye structures. The third principle is to treat the elevated intraocular pressure once established and the last one is to control the inflammatory process. Anyway, the best therapy is prevention and in cases with neovascular glaucoma, this strategy is the most preserving for visual acuity. It becomes critical to examine thoroughly all the suspects for neovascular glaucoma, patients with risk factors, especially those with proliferative diabetic retinopathy and central retinal vein occlusion. Another significant aspect is to be very attentive during clinical examination even if the intraocular pressure is normal. In cases of iris and/ or iridocorneal angle neovascularization, panretinal photocoagulation and anti-VEGF therapy will be started immediately. Until the neovascular glaucoma is established, we could cope with the neovascularization, and treatment can delay its onset [**2**,**26**,**27**].

**Table 4** highlights some therapeutic strategies in various stages of neovascular glaucoma.

**Tabel 4:** Therapeutic strategies in various stages of neovascular glaucoma [**2**,**44**-**47**]

Stage	Description	Ocular features			Treatment	
			PRP	Anti-VEGF	Anti-glaucoma medication	Glaucoma filtration surgery
I	Preglaucoma	Neovascularization of iris	Yes	Yes	No	No
II	Open-angle	Elevated IOP, neovascularization of the ICA	Yes	Yes	Yes	Yes/ No
III	Closed-angle	Elevated IOP, neovascularization of the ICA	Yes	Yes	Yes	Yes

## Panretinal photocoagulation (PRP)

Panretinal photocoagulation is still the gold-standard for the treatment of neovascularization. Every time the retina is subjected to an ischemic process, it should be considered for treatment. Usually, PRP needs one to three sessions, with spot diameter about 1200 to 1600 microns. In patients with neovascular glaucoma, these sessions will be performed as quickly as possible and it does not matter if there is just iris rubeosis or an advanced stage of NVG with peripheral anterior synechiae. PRP can be performed if ocular fundus has a good visibility. If there is a poor view of the fundus, anterior retinal cryotherapy (ARC) and ARC in association with anti-VEGF may serve as an option. Moreover, in extreme cases, vitrectomy could be associated with anti-VEGF injection, panretinal photocoagulation and endocyclophotocoagulation.

Panretinal photocoagulation is recommended in patients with ocular ischemic syndrome, anterior and posterior segment of the eye neovascularization, in order to prevent the development of neovascular glaucoma. PRP should be performed very carefully because if used as monotherapy it can contribute to IOP elevation and will affect the vascularization of the optic nerve. In addition to its destructive action on ischemic tissue, PRP can also destruct some normally functioning tissues. It is clinically meaningful that after PRP the patient with elevated IOP will continue to administer the necessary topical hypotensive agents until the PRP effect establishes. This can happen during some weeks, and equivalate with the regression of new vessels [**29**,**48**-**51**].

## ANTI-VEGF therapy

The anti-VEGF era opened a new therapeutic horizon for different conditions. It was used for the first time in 2006, when a series of medical institutions started to use anti-VEGF worldwide. The following can be mentioned as anti-VEGF remedies used in the treatment of neovascular glaucoma: bevacizumab (BVZ (Avastin; Genetech, South San Francisco, CA, USA)), ranibizumab (RBZ (Lucentis; Genetech, South San Francisco, CA, USA)), conbercept (Lumitin, Chengdu Kanghong Biotech Company), aflibercept (AFB (Eylea; Regeneron Pharmaceuticals, Tarrytown, NY, USA)), brolucizumab (Beovu®; manufactured by Novartis).

**Bevacizumab** is a full-length, humanized monoclonal antibody directed against all the biologically active isoforms of VEGF-A. It has a molecular weight of approximately 149 kilodaltons (kD). In 2004, U.S. FDA approved BVZ for treatment of colorectal cancer and in 2008 for breast cancer. BVZ is still used off-label against ocular conditions.

**Ranibizumab** is a recombinant humanized IgG1 monoclonal antibody fragment that binds to and inhibits vascular endothelial growth factor A (VEGF-A). RBZ is a 48-kD Fab fragment, comparatively to a full-length BVZ. It was first approved by the FDA in 2006 for wet age-related macular degeneration. Since then, it has been approved for the treatment of macular edema following retinal vein occlusion, diabetic macular edema, and choroidal neovascularization in pathological myopia. Most recently, it was approved in 2015 for patients with diabetic retinopathy.

**Conbercept** is a new generation anti-VEGF, a 100 percent humanized fusion protein that targets VEGF-B, placental growth factor (PIGF), and various isoforms of VEGF-A. In China, it was approved for the treatment of neovascular AMD in 2014, for the treatment of pathological myopia associated with choroidal neovascularization in 2017, and for the treatment of diabetic macular edema in 2019. As of December 2020, conbercept is undergoing phase III clinical trials through the U.S. Food and Drug Administration’s PANDA-1 and PANDA-2 development programs.

**Aflibercept** is a 115 kDa fusion protein, a soluble decoy receptor that binds vascular endothelial growth factor-A (VEGF-A), VEGF-B and placental growth factor (PIGF). AFB has a greater affinity than the body’s native receptors. It is called a decoy receptor as VEGF does not bind to its original receptors and mistakenly binds with aflibercept, thereby reducing VEGF’s activity. Comparing to BVZ, AFB forms stable, inert, and homogenous immune complexes with VEGF. BVZ-VEGF immune complexes are heterogenous and multimeric. So, AFB does not induce the platelet adhesion or deposition in the systemic circulation. Aflibercept was approved by the FDA in 2011 for the treatment of neovascular (wet) age-related macular degeneration, in 2014 for the treatment of diabetic macular edema (DME) and in 2015 for the treatment of diabetic retinopathy in patients with DME. Also in 2015, AFB was approved by the European Commission for the treatment of macular edema secondary to retinal vein occlusion, both central and branch.

**Brolucizumab** is a humanized monoclonal single-chain variable fragment (scFv) that binds and inhibits vascular endothelial growth factor A (VEGF-A). Brolucizumab is currently FDA-approved for the treatment of neovascular age-related macular degeneration (AMD/ ARMD). It was approved in October 2019 and is currently the only approved single chain antibody fragment. In June 2020, an updated label that included the adverse events of retinal vasculitis and retinal vascular occlusion was included by the FDA. Brolucizumab has the lightest molar mass (26 kD) of any treatment currently available, which means a higher concentration of molecules that is delivered to the retinal tissue. In addition, smaller molecules are thought to penetrate the retinal tissue to the choroid more effectively [**14**,**31**,**32**,**52**-**58**].

There are some studies that investigated the bevacizumab, ranibizumab and aflibercept effect in patients with neovascular glaucoma. These agents were administrated topically, in the anterior chamber or in the vitreous body. Waisbourd et al. presented a pilot study about the bevacizumab efficacy. When instilled topically in 8 patients, BVZ 25 mg/ ml, 4 times per day, for two weeks, the average IOP reduction was about 6,1 mmHg. Three patients presented a regression of the iris neovessels. Injected into the anterior chamber, BVZ reduced the necessity of surgery in NVG patients. A separate study showed less iris neovessels leakage one day after injection of bevacizumab into the anterior chamber. Grover et al. reported a considerable lowering of VEGF concentration in the aqueous humor after injecting BVZ into the anterior chamber. Lüke et al. published a prospective study, trying to analyze the efficacy of ranibizumab (0.5 mg/ 0.05 mL) injected into the eye in ten patients with neovascular glaucoma. It has been proved that RBZ has a good anti-angiogenic outcome and can prevent or even stop the closing of the iridocorneal angle. A recent study showed that injection of aflibercept (2 mg) into the vitreous body of four patients with neovascular glaucoma (stage 1 or 2) is efficient for iris and iridocorneal regression of neovessels, and IOP stabilization or lowering. AFB was administered at first visit, at four and eight weeks and then at each 8 weeks during 52 weeks [**14**,**59**-**65**]. It is indispensable to know that after the regression of neovessels, the iridocorneal angle appears open at gonioscopy, but ghost vessels, which are transparent and tend to form synechiae adhesions with further closing of the angle, may be seen. These patients are recommended antiglaucoma surgery, this ensuring a better IOP control and prevention of the glaucomatous optic neuropathy. Anti-VEGF therapy assumes a better visual prognosis and a better controlled IOP in patients with neovascular glaucoma, by reducing neovascularization. Unfortunately, its effects are temporary and last for four to six weeks and anti-VEGF cannot work on the fibrovascular membrane that closes the iridocorneal angle. It is a challenge for further studies to find the best way for anti-VEGF administration, to choose the right dose and the right way, timing, association with other agents, to prolong its action and use some intraocular delivery systems. Another strategy is to study the effects of new therapies in order to target other VEGF types, to use anti-TGF-beta and anti-HGF remedies [**2**,**4**,**5**,**25**,**31**-**33**,**66**].

## Drug therapy

Antiglaucoma medications aim to reduce the elevated intraocular pressure and to ameliorate the patient’s comfort. For this reason, some ocular hypotensive drugs are used: carbonic anhydrase inhibitors (both oral and topical), beta-blockers, and alpha-2 agonists, which lower aqueous production and possibly ameliorate its outflow. Prostaglandin analogs and anticholinergic agents (like pilocarpine) should be avoided because they may aggravate the inflammation. Anyway, prostaglandin analogs are effective and are used only when IOP cannot be controlled with other medications, but in patients with neovascular glaucoma they are less effective. Miotics should also be avoided because they may worsen synechial angle closure by anterior displacement of the iris-lens diaphragm and blocking the aqueous humor outflow from the anterior chamber. Topical steroids and cycloplegics help treating inflammation and are considered supportive measures in patients with neovascular glaucoma. Also, steroids reduce the vascular permeability and angiogenesis, and patients feel more comfortable. Other temporary measures to reduce the elevated IOP are hyperosmotic agents like mannitol and glycerol, administered orally or systemically [**9**,**67**,**68**].

## Surgical treatment

Surgical treatment is another claiming topic for the surgeon, because the neovascular glaucoma is refractory to any intervention and the anatomy of iridocorneal angle is modified by proliferated tissues. After all the non-invasive strategies were applied to treat the NVG, but they were ineffective, surgery becomes the only solution to achieve the target IOP. Antiglaucoma surgery includes: trabeculectomy (glaucoma filtration surgery is the gold standard), glaucoma drainage devices (GDDs) and cyclodestructive procedures (cyclophotocoagulation and cyclocryotherapy). 

*Traditional trabeculectomy*

Traditional trabeculectomy, also known as glaucoma filtration surgery, is the first type of glaucoma surgery and is the less efficient. Its failure happens because of the high rate of severe inflammation and hyphema. 

*Trabeculectomy with antimetabolites and antifibrotics*

As already discussed, in cases of poor view of the fundus, anterior retinal cryotherapy (ARC) and ARC in association with anti-VEGF or vitrectomy associated with anti-VEGF injection, panretinal photocoagulation and endocyclophotocoagulation may be performed. After this, trabeculectomy with antimetabolites and antifibrotics use presented a high rate of success. Among the antimetabolites, mitomycin C (MMC) or 5-fluorouracil (5-FU) can be used. Trabeculectomy with MMC has an efficiency of 62,6% to 81,2% in the first year after surgery and decreases gradually to 51,3% at five years. It is an interesting fact that injection of bevacizumab in the vitreous body before trabeculectomy with MMC, reduces the risks of postoperative hyphema and improves the surgery outcome.

To better understand the origins of traditional trabeculectomy failure, we should assess some important aspects of the postoperative wound healing process. During wound healing, including the postoperative period in glaucoma surgery, a highly coordinated and synchronized series of key events take place. The first step in wound healing is rapid hemostasis. The second is the inflammatory reaction. The third is cellular differentiation. The fourth – proliferation and migration of cells to injured place. The fifth is controlled angiogenesis. The sixth – wound epithelialization and the seventh – synthesis and arrangement of collagen fibers in a specific way to fix the healing tissue. All these lead to an aggressive healing of fistula at the level of episclera and conjunctiva, blocking the drainage with collagen, neovessels and fibroblasts. The IOP do not reduce as it was supposed and the surgery fails. The secret for a good, functional filtration bleb is to create conditions for it to be avascular, because new vessels are a sign for poor prognosis. So, we must interrupt some key steps of wound healing. For this reason, we can apply 5-FU and MMC, which inhibit the vascularization and are associated with a better postoperative outcome. In addition to their efficiency, these antimetabolites can also excessively injure the underlying tissues and tend to induce some complications, such as postoperative hypotony, corneal toxicity, a very thin wall of the filtration bleb that can allow an overdrainage of the aqueous humor and is an opportune way for the infection to penetrate the eye. Because of these disadvantages, in the last years, there have been some researches on finding other remedies to modulate the wound healing process. Among them is the anti-VEGF effect, especially on the fifth step, angiogenesis. It is also known that VEGF in produced by endothelial cells, macrophages, fibroblasts, platelets, neutrophils, and smooth muscle cells. All of them participate in the wound healing process. Besides the angiogenesis, VEGF also stimulates epithelialization and collagen deposition. There are some studies confirming that high concentrations of VEGF were found in the aqueous humor and Tenon tissue of patients with failed trabeculectomy, compared with patients in whom the filtration worked. This phenomenon lasts for about one year postoperatively. So, a good strategy is to associate glaucoma filtration surgery with anti-VEGF agents [**2**,**69**-**73**].

*Glaucoma drainage devices (GDDs) and minimally invasive glaucoma surgery (MIGS)*

In cases of failed trabeculectomy and when a good postoperative outcome is impossible to obtain because of conjunctival scaring, it becomes relevant that the implantation of glaucoma drainage devices is a must. There are two main types of GDDs: 1) valved or flow restrictive implants (like Ahmed glaucoma valve (AGV)) and 2) nonvalved implants (like Baerveldt, Molteno, and Aurolab aqueous drainage implant (AADI)). Valved implants are preferred for neovascular glaucoma patients because they can reduce IOP immediately, are associated with a lower risk of ocular hypotony and iris damage after surgery. The first attempts to create a glaucoma drainage device were in 1906, although the first type of shunt appeared in 1976. It was the Molteno shunt (Molteno Ophtalmic Limited, Dunedin, New Zeeland and IOP Inc. Costa Mesa CA, USA), created by C.B. Molteno et al. and consists of a thin silicone tube, which drains the aqueous humor at the level of an episcleral plate. The episcleral plate is covered by the Tenon fascia and conjunctiva, and forms a unilocular and circular space with a fibrovascular capsule. This controls the discharge of aqueous humor from the eyes and maintains IOP at a certain level. Because of a high postoperative complication rate like: ocular hypotony, the shallowing of anterior chamber, choroidal effusions, and choroidal detachment, it becomes imperative to create valved implants. In 1976, Krupin proposed a unidirectional valve, sensitive to IOP changes, which opens at IOP more than 11 mmHg. Later, other implants like Baerveldt (Advanced Medical Optics, Inc. Santa Ana, CA, USA), Shocket, Eagle Vision (Eagle Vision, Inc. Memphis, TN, USA) and Ahmed valve (New World Medical, Rancho Cucamonga, CA, USA) were created and became available. Ahmed glaucoma valve has a better mechanism to control the IOP. It was created by Mateen Ahmed and approved by FDA US in 1993. Ahmed valve has three main parts: 1) a plate, in medical grade silicone, polypropylene, or porous polyethylene, depending on the model; 2) a drainage tube in medical grade silicone; and 3) a valve mechanism in medical grade silicone. Nowadays, there are at least eleven available models of Ahmed valve (single and double plate, pars plana and pars plana pediatric, and others). There are studies confirming that GDDs are very useful in cases of refractory glaucoma. Nevertheless, the postoperative risks and complications remain actual and patients need special care 2 to 3 months after surgery. In this context, the minimally invasive glaucoma surgery (MIGS) was developed, a real revolution in ophthalmology, which has been more and more preferred in the last years and some authors call it “The new age of glaucoma surgery”. MIGS are a safer option to reduce the intraocular pressure than conventional surgery, with the added benefits of a higher success rate and a faster recovery time, even if they are a more expensive technique. There are three different categories of MIGS according to the way they decrease intraocular pressure: 1) MIGS that drain the aqueous humor into the subconjunctival space (aqueous shunt, Ex-PRESS shunt, XEN gel stent, InnFocus Microshunt), 2) MIGS that drain AH through the trabecular meshwork (iStent, iTrack, Hydrus, Trabectome/ Trab 360, OMNI 360) and 3) MIGS that drain the AH in the suprachoroidal space (CyPass Micro-stent).

*Some interesting data*

In a retrospective study of 44 eyes with neovascular glaucoma, the short-term efficacy and safety of the Ex-PRESS shunt were appreciated compared with trabeculectomy. 14 eyes underwent Ex-PRESS implantation and 30 – trabeculectomy. In all cases of prominent rubeosis iridis, panretinal photocoagulation and intravitreal injection of 1,25 mg bevacizumab were done three days before surgery. At one-year follow-up, patients with Ex-PRESS shunt had less IOP reduction, but the complication rate in them was low, as it is a safer method than trabeculectomy. Liu et al. studied the efficacy and safety of trabeculectomy (with intravitreal injection of ranibizumab) compared to Ahmed valve implantation. 37 eyes of 36 patients with neovascular glaucoma were included in their prospective study. 18 eyes underwent RBZ injection into the vitreous body seven days before trabeculectomy. 19 eyes had AGV implantation. Patients with combined therapy showed a better outcome, a significant IOP reduction, improvement of best corrected visual acuity and a lower complication rate. Olmos et al. presented a retrospective study of 163 eyes of 151 patients with neovascular glaucoma. The standard antiglaucoma therapy was applied in all of them. In addition, 64 eyes had intravitreal injection of bevacizumab, and it delayed the necessity of surgical treatment. PRP also delayed this term, but in a better way than BVZ. In their non-randomized, prospective study, Tang et al. investigated 43 eyes of 43 patients with neovascular glaucoma. 21 of them had 0,5 mg ranibizumab intravitreal injection 3-14 days before the Ahmed valve implantation. In 22 the AGV was implanted without anti-VEGF. At six-months follow-up, the results were quite similar, the success rate in group with RBZ was 73,7% and 71,4% in the AGV group. After one year, the first group showed a 72,2% and the second – 68,4% success rate. So, they concluded that there is no significant difference between study groups with NVG, neither in IOP, nor in best corrected visual acuity (BCVA). Sahyoun et al. presented a retrospective study of long-term outcomes of Ahmed valve implantation in association with intravitreal bevacizumab injection in patients with neovascular glaucoma. The study included 39 eyes of 34 patients with NVG. The first group, of 19 eyes, underwent BVZ seven days before surgery and the second one – 20 eyes, had no BVZ. As a result, BVZ injection did not improve the IOP reduction or BCVA outcome, but reduced a lot the risk of postoperative hyphema. The same results were presented by Zhou et al. in their separate study. The Tube Versus Trabeculectomy (TVT) Study is a multicenter randomized clinical trial that compares the safety and efficacy of tube shunt surgery to trabeculectomy with mitomycin (MMC) in eyes with previous cataract and/ or failed glaucoma surgery. Surprisingly, both methods showed good postoperative outcome. There was no significant difference in the rate of vision loss following trabeculectomy with MMC and tube shunt surgery after 1 year of follow-up. Cataract progression was common, but occurred with similar frequency with both surgical procedures.

Very often, eyes with very poor visual potential, with recurrent and refractory IOP elevation need cyclodestructive procedures. The most popular are transscleral cyclophotocoagulation and laser endocyclophotocoagulation. The success rates of micropulse cyclophotocoagulation (MP-CPC) were 75% after 3 months, 66% after 6 months and 67% at last visit (Williams et al. study). Seven patients with MP-CPC presented ocular hypotony (8,8%), 21 patients had prolonged anterior chamber inflammation (26%, 1+ flare more than 3 months), 13 patients lost more than 2 rows of BCVA in 3 months (17%), 4 patients had diabetic macula edema (5%), 2 patients with corneal edema and another 2 with ocular phthisis bulbi. Besides the drug, laser, and surgical treatments, neovascular glaucoma progresses and leads to total blinding, which is associated with violent pain. In these cases, the injection of alcohol into the retrobulbar space should be considered as a therapeutical method. Sometimes, evisceration or enucleation are the only reasonable decisions [**2**,**9**,**13**,**51**,**74**-**85**].

## Conclusions

1. Neovascular glaucoma is a type of secondary glaucoma; in 95% of cases, it is caused by retinal ischemia, associates neovessels in iris, iridocorneal angle and elevated intraocular pressure. It is refractory to treatment and has a high rate of severe visual impairment. 

2. There are three most common causes of neovascular glaucoma: diabetic retinopathy (in 33% of cases), ischemic retinal vein occlusion (33%) and ocular ischemic syndrome (13%).

3. The treatment of neovascular glaucoma is a real challenge both for the clinician and the patient. Very often, even if the necessary therapy is applied in a correct way and in good terms, the patient’s outcome and visual prognosis are poor and his quality of life is affected severely.

4. The best therapy is prevention. It is critical to examine thoroughly all the suspects for neovascular glaucoma, patients with risk factors, especially those with proliferative diabetic retinopathy and central retinal vein occlusion.

5. The anti-VEGF era improved the patients’ outcome. These agents reduce neovascularization, but they have a temporary action and their effect lasts for 4-6 weeks, also, they cannot work on the fibrovascular membrane.

6. There is a chance for neovascular glaucoma to be treated by using an etiopathogenetic approach. For this reason, further studies are necessary and medical community is searching for new therapies. In addition to anti-VEGF therapy, there are a lot of pro-angiogenic factors, whose inhibition may improve the results. It is a long, tremendous path, full of challenges and technical difficulty, but we are waiting for its successes to help the neovascular glaucoma patients and protect them from blinding.

**Conflict of Interest statement**

The authors state no conflict of interest.

**Acknowledgements**

None.

**Sources of Funding**

None.

**Disclosures**

None.
